# Adult gaucher disease in southern Tunisia: report of three cases

**DOI:** 10.1186/1746-1596-7-4

**Published:** 2012-01-10

**Authors:** Faten Ben Rhouma, Faten Kallel, Rym Kefi, Wafa Cherif, Majdi Nagara, Hela Azaiez, Ines Jedidi, Moez Elloumi, Sonia Abdelhak, Sondes Mseddi

**Affiliations:** 1 Molecular Investigation of Genetic Orphan Diseases, Pasteur Institute, Tunis - Tunisia; 2 Child Neurological diseases Unit, Faculty of Medicine, Tunis- Tunisia; 3 Hematology Department, Hedi Chaker Hospital, Sfax- Tunisia

**Keywords:** Adult, Gaucher disease, p.N370S, Parkinsson disease, Tunisia

## Abstract

**Background:**

Gaucher disease (GD) is the most frequent lysosomal storage disorder; type 1 is by far the most common form. It is characterized by variability in age of onset, clinical signs and progression. It is usually diagnosed in the first or second decade of life with the appearance of bone pains, splenomegaly and thrombocytopenia, but the disease may be diagnosed at any age between 1 and 73 years. In the present study, we report 3 cases with late onset of GD in whom the disease was a surprise finding including one patient with Parkinson disease. This late onset is described as an adult form of Gaucher disease.

**Findings:**

Molecular investigation showed mutational homogeneity in Tunisian adult patients suffering from GD. Indeed, all patients carry the p.N370S mutation: two patients at a homozygous state and one patient at compound heterozygous state.

**Conclusion:**

The p.N370S mutation presents a large variability in the onset of the disease and its clinical manifestation supporting the view that GD should be considered as a continuum phenotype rather than a predefined classification.

## Findings

Gaucher disease (GD) is the most frequent lysosomal storage disorder [1]. It is an autosomal recessive inborn defect in the glucocerebrosidase gene (*GBA*) on1q21 leading to enzymatic deficiency of the β-glucocerebrosidase and the accumulation of glycosylceramide substrate in the macrophage's lysosomes. GD is characterized by a considerable phenotypic and genotypic heterogeneity [2]. There is a wide range of clinical manifestationsfrom nearly asymptomatic patients who have mild bone pain up to full spectrum of severe complications with cytopenia, hepatosplenomegaly and pathologic fractures [3]. More than 200 mutations were identified along *GBA *gene http://www.hgmd.org. In previous studies, investigation of GD showed that p.N370S, p.L444P and Rec*Nci*I mutations were relatively frequent in Tunisian patients [4,5]. In the present study, we report three Tunisian adult GD patients including one patient with Parkinson disease in whom the disease was incidentally found following bone marrow examination during check up for thrombocytopenia with splenomegaly. These cases were investigated for the p.N370S, p.L444P and Rec*Nci*I mutations in *GBA *gene.

Three patients diagnosed with GD from three unrelated families from southern Tunisia were investigated. The diagnosis was based on the occurrence of hepatosplenomegaly associated with hematological abnormalities and/or bone lesions and was confirmed in all patients by the presence of Gaucher cells.

After the patient's written informed consent, 5 ml of blood were collected. DNA was extracted from peripheral blood mononuclear cells using the standard salting-out procedure. DNA samples were then amplified by polymerase-chain-reaction (PCR) followed by enzymatic restriction using *Xho*I and *Nci*I to screen for p.N370S and p.L444P respectively. Sequencing was performed on an ABI 3130 to detect the three mutations (p.N370S, p.L444P and Rec*NciI*).

### Case 1

**Figure 1:**
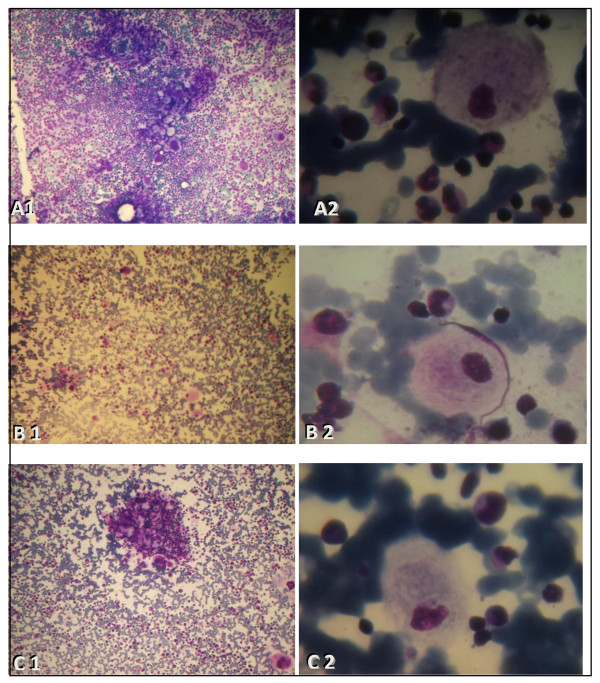
**Micorscopic images of Gaucher cells of the three studied GD patients: A cell with a ''sandpaper'' appearance was evident**. A: Myelogram of Case 1: microscopic image of Gaucher cells (A1: MGG, X10) and (A2: MGG, X100). B: Myelogram of case 2: microscopic image of Gaucher cells (B1: MGG, X10) and (B2: MGG, X100). C: Myelogram of Case 3: microscopic image of Gaucher cells (C1: MGG, X10) and (C2: MGG, X100). *Abbreviation: MGG; May-Grünwald Giemsa*.

A 46 years old male was referred to the hematology departement of Sfax in Southern Tunisia for thrombocytopenia (platelet 20 000/mm^3^). Clinical examination showed a voluminous splenomegaly associated with pancytopenia. Neurological symptoms were absent. The diagnosis of GD was confirmed by bone marrow examination (Figure 1: A1, A2). The patient underwent partial splenectomy. Spleen and liver histology showed Gaucher cells infiltration. The patient did not follow any specific enzymotherapy. At the age of 51, he started suffering from pain in his left leg with a complete functional impairment. Radiological investigations of the leg showed an Erlenmeyer flask deformity and cortical fracture requiring surgical treatment. A bone biopsy was performed and revealed the presence of Gaucher cells. The patient was treated with a cast immobilization and an antalgic symptomatic treatment. The treatment outcome showed a limited effectiveness, with no consolidation and persistence of bone pain. An osteosynthesis by external fixator was then performed. The patient developed Parkinson disease presented with ataxia, rigidity and tremor at the age of 52. Family history was negative for GD although one brother suffered from Schizophrenia. Screening of exons 9 and 10 of *GBA *gene revealed that the patient was compound heterozygous for the p.N370S and Rec*Nci*I mutations.

### Case 2

A 35 years old female was referred to the hematology departement of Sfax with thrombocytopenia (platelet count 100 000/mm^3^). Family history showed no particular pathologies. The diagnosis of GD was confirmed by a myelogram and bone marrow biopsy that revealed the presence of Gaucher cells (Figure 1: B1, B2). Subsequently, biochemical analysis of β-glucocerebrosidase activity in leucocytes confirmed enzyme deficiency. Neurological and osteoarticular examination were normal. Molecular genetic testing for the three common Gaucher mutations revealedhomozygosity for the p.N370S mutation. The patient being monitored regularly and is undergoing a therapeutic trial with a substrate reduction therapy initiated on April 2011.

### Case 3

The patient is a 34 year-old female from a consanguineous family. Her mother has developed uterine fibroid, two sisters had cholecystectomy and one brother had a bone disease. Her husband and her two children are in a good health. Her initial clinical presentation was an unexplained bicytopenia (white blood cells of 2905/mm^3 ^and platelet count 130 000/mm^3^) and splenomegaly. The diagnosis of GD was confirmed by myelogram (Figure 1: C1, C2). Spleen histology showed Gaucher cells. Molecular analysis including PCR/RFLP and DNA sequencing identified the p.N370S mutation at the homozygous state. The patient underwent a partial splenectomy and the outcome showed improvement of pancytopenia.

## Discussion

To the best of our knowledge this is the second report describing adult patients with GD in Tunisia and the first report of an association of GD and Parkinson disease in our Country. The first report by Dandana et al [6] described a 50 year old patient with GD without any neurological disorder. Many adult cases with GD have been reported worldwide. Based on data from the Gaucher Registry (ICGG 2008) the p.N370S mutation was found in 53% of all GD patients and represents the highest prevalence. About 14% of GD patients were diagnosed between the age of 31 to 50 year old [7].

Among the patients we report here, two patients were homozygous for the p.N370S mutation (table 1). Homozygosity for this mutation appears common among GD type I adult patients [8]. So far, this mutation was found in 44% of paediatric Tunisian patients with GD [5]. Unlike most of the autosomal recessive genetic diseases in Tunisia for which the majority of the patients is born to consanguineous marriages and is homozygous for deleterious alleles, the p.N370S mutation is encountered at heterozygous state in combination with other mutated alleles [5].

**Table 1:** Clinical and genetic features of 3 Tunisian GD patients.

Patients	Sexe	Consanguinity	Clinical findings						Genetic status
				
			*Onset age (year)*	*HMG*	*SMG*	*Platelet level*	*Neurological symptoms*	*Osteoarticular* *symptoms*	
***Case 1***	M	No	46	-	+	20 000/mm^3^	+	+	N370S/RecNciI
***Case 2***	F	Yes	35	-	+	100 000/mm^3^	-	-	N370S/N370S
***Case 3***	F	Yes	34	-	+	130 000/mm^3^	-	-	N370S/N370S

Abbreviations: HMG, hepatomegaly; SMG, splenomegaly; M, male; F, female

One patient (case 1) developed Parkinson disease at age 52. The co-morbidity of GD and Parkinson disease has been largely discussed [9-11]. The incidence of Parkinson disease seems particularly high among patients with GD; Bembi and collaborators found four Parkinson patients (6.9%) among 58 patients with GD [12]. Lachmann and Platt found 3 cases of Parkinson disease in 130 patients (2.3%) [13]. The first signs of Parkinson disease appear at an earlier age in patients with GD compared to the general population: 48 versus 71 years average age, respectively [14]. It has been increasingly recognized that Parkinsonism may be a clinical feature of GD type I and may even precede its diagnosis [15]. In addition, heterozygosity for certain *GBA *gene mutations predisposes to a higher risk for Parkinson disease according to Chérin and collaborators [16]. Whereas, a recent study presented by Nishioka and collaborators on 395 patients with Parkinson disease and 372 control subjects did not show a statistically significant association with p.K26R, p.K186R and p.N370S in the Tunisian population (P > 0.05) [11]. The p.N370S mutation was identified in only one sporadic patient with Parkinson disease and 3 control subjects indicating that the frequency of this mutation in Tunisian patients is much lower than that observed in Ashkenazi Jews patients with Parkinson disease (31%).Furthermore, Spitz and collaborators reported that most patients with GD never develop Parkinson disease, suggesting the involvement of other genetic and/or environmental factors in the GD process [15].

It has been suggested that only one third of the patients who were homozygote for p.N370S mutation come to medical attention and that about two thirds remain asymptomatic throughout life [17]. Carrier screening programs may lead to the identification of asymptomatic cases. In the literature, carrier screening and termination of pregnancy of GD is controversial, in particular for mild mutations like the p.N370S mutation [18,19]. In Tunisia, GD like other autosomal recessive disorders [20,21], is rare and does not represent a major public health concern. We suggest that prenatal diagnosis for couples at risk carrying a severe mutation (L444P, Rec*Nci*I) is the best alternative to reduce the incidence of the severe form of the disease.

There is a great variability in the clinical manifestation as well as in the onset and course of GD. This supports the need for a new classification and reinforces the hypothesis of the concept of a phenotypic continuum. Clinicians, especially in hematology and internal medicine, should suspect the diagnosis of GD in any adult patient suffering from abdominal and/or bone pain with unexplained hematological disorder especially in combination with organomegaly.

## Abbreviations

GD: Gaucher disease; PCR: polymerase chain reaction; PD: Parkinson disease; RFLP: Restriction fragment length polymorphism.

## Competing interests

The authors declare that they have no competing interests.

## Authors' contributions

SA had full access to all the data in the study and takes responsibility for the integrity of the data and the accuracy of the data analysis. Study concept and design: SA and SM. Acquisition of data: FBR, FK, RK, WC, MN, HA, IJ and ME. Analysis and interpretation of data: FBR, RK, WC, MN, HA, SA and SM. Drafting the manuscript: FBR. Critical revision of the manuscript for important intellectual content: SA, RK, HA, SM. Obtained funding: SA. Administrative, technical or material support: FBR, RK, WC, MN, and HA. Study supervision: SA, ME and SM. All authors read and approved the final manuscript.
